# YWHAG overexpression correlates with an immune-desert microenvironment and poor prognosis in laryngeal squamous cell carcinoma

**DOI:** 10.3389/fimmu.2026.1936961

**Published:** 2026-09-15

**Authors:** Naoto Adachi, Yuki Sonoda, Fusako Takezawa, Yukihiro Kimura, Shohei Higuchi, Yohei Sato, Masafumi Kanno, Shigeharu Fujieda

**Affiliations:** 1 Department of Otorhinolaryngology Head and Neck Surgery, University of Fukui, Fukui, Japan; 2 Department of Pathology, Faculty of Medical Sciences, University of Fukui, Fukui, Japan; 3 Immunology and Allergy Research Unit, Department of Otorhinolaryngology Head and Neck Surgery, University of Fukui, Fukui, Japan

**Keywords:** B cells, CIBERSORT, laryngeal squamous cell carcinoma, tertiary lymphoid structures, tumor immune microenvironment, YWHAG

## Abstract

**Background:**

Laryngeal squamous cell carcinoma (LSCC) remains a major cause of cancer-related mortality among head and neck malignancies. The tumor immune microenvironment plays a critical role in determining disease outcome; however, the specific immune cell composition and its prognostic relevance in LSCC have not been fully elucidated.

**Methods:**

CIBERSORT was applied to bulk RNA-sequencing data from 381 TCGA-HNSCC samples to estimate the relative fractions of 22 immune cell types. LSCCs (n = 64) were selected for in-depth analyses. Consensus clustering based on immune profiles stratified patients into three clusters. Differentially expressed genes (DEGs) and pathway enrichment analyses were performed between Cluster 1 (immune-desert) and Clusters 2 & 3 (immune-active). Kaplan–Meier survival analysis was used for prognostic assessment. YWHAG protein expression was evaluated by immunohistochemistry in an independent institutional cohort of patients with LSCC (n = 23), and its correlation with clinicopathological parameters was examined.

**Results:**

Across HNSCC subsites, LSCC tumors exhibited significantly higher infiltration of naive B cells and plasma cells than tumors from other anatomical sites (Kruskal-Wallis test, adjusted *P* < 0.05). Consensus clustering of LSCC identified three distinct immune profiles: Cluster 1 (immune-desert, dominated by M0 macrophages and eosinophils), Cluster 2 (immune-hot, enriched for B cells, plasma cells, and CD8+ T cells), and Cluster 3 (mixed-inflammatory). Tertiary lymphoid structure (TLS)-related genes were uniformly and significantly downregulated in Cluster 1 (*P* < 0.05). Furthermore, pathway analysis revealed that B cell-related pathways were significantly suppressed in Cluster 1. DEG analysis within Stage I–III tumors revealed *YWHAG* as the sole gene significantly upregulated in Cluster 1. High *YWHAG* expression was associated with significantly shorter overall survival across all stages (log-rank *P* < 0.05). In the institutional immunohistochemistry cohort, strong YWHAG staining was significantly associated with larger tumor size.

**Conclusions:**

LSCC harbors distinct immune microenvironmental subtypes. Tumors enriched for B cell- and TLS- related gene signatures demonstrate favorable outcomes, whereas immune-desert tumors with elevated *YWHAG* expression are associated with poor prognosis. YWHAG expression may be associated with unfavorable outcomes, possibly due to reduced antitumor immune responses and increased tumor size.

## Introduction

Head and neck squamous cell carcinoma (HNSCC) encompasses malignancies arising from the oral cavity, oropharynx, larynx, and hypopharynx, collectively representing the sixth most common cancer worldwide (1–3). Laryngeal squamous cell carcinoma (LSCC) accounts for approximately 25–30% of HNSCC cases and carries a five-year overall survival (OS) rate of approximately 60% for early-stage disease, declining sharply to below 40% for advanced-stage tumors despite multimodal treatment (4, 5). Beyond surgical resection and radiotherapy, immune checkpoint inhibitor therapy targeting the PD-1–PD-L1 axis has received regulatory approval for recurrent or metastatic HNSCC; however, durable responses are observed in only a subset of patients, highlighting the urgent need for reliable predictive and prognostic biomarkers (6, 7).

The tumor immune microenvironment plays a central role in governing tumor progression, immune evasion, and treatment responsiveness (8–10). Recent transcriptomic studies have revealed substantial heterogeneity in immune cell composition across HNSCC subsites (11–14); yet the systematic characterization of the immune landscape specifically within LSCC remains limited (15). CIBERSORT, a state-of-the-art computational deconvolution algorithm, enables the quantification of 22 distinct immune cell populations from bulk RNA-sequencing (RNA-seq) data with high accuracy (16). Tertiary lymphoid structures (TLS) are ectopic lymphoid organs that develop within tumors and orchestrate adaptive antitumor immunity through the colocalization of B cells, T cells, and dendritic cells (17–20). Emerging evidence consistently demonstrates that intratumoral TLS formation, particularly marked by B cell-related gene signatures and *CXCL13* expression, correlates with favorable outcomes in multiple solid tumor types, including HNSCC (21). However, the contribution of TLS to prognosis specifically in LSCC has not been investigated. *YWHAG* (14-3–3 gamma) encodes a ubiquitous scaffold protein belonging to the 14-3–3 family, which modulates diverse oncogenic pathways encompassing cell cycle regulation, apoptotic resistance, and receptor tyrosine kinase signaling (22, 23). *YWHAG* upregulation is an adverse prognostic factor in lung, gastric, and hepatocellular carcinomas (24–27). However, its biological role and clinical significance in LSCC have not been elucidated (28, 29).

In this study, we performed large-scale immune deconvolution of The Cancer Genome Atlas (TCGA)-HNSCC and conducted LSCC-specific analyses that revealed a unique B cell-enriched immune profile, three immune phenotypes with distinct immune-cell compositions, and the novel association of *YWHAG* overexpression with an immune-desert phenotype. We subsequently assessed YWHAG protein expression using immunohistochemistry in an independent institutional cohort, demonstrating its correlation with adverse pathological parameters. These findings collectively support *YWHAG* may be associated with unfavorable outcomes.

## Materials and methods

### TCGA data acquisition

RNA-seq data and their clinical annotations were obtained from the TCGA Genomic Data Commons (GDC) data portal for the TCGA-HNSC project (n = 381, https://portal.gdc.cancer.gov/). To exclude potential confounding effects of human papillomavirus, oropharyngeal carcinomas were excluded from the analysis. Samples with missing survival data were excluded from Kaplan–Meier analyses.

### Immune cell deconvolution using CIBERSORT

Immune cell fractions were estimated from bulk RNA-seq data using the CIBERSORT algorithm, enabling the quantification of 22 distinct immune cell subtypes (16). Transcripts per million (TPM)-normalized expression matrices derived from TCGA-HNSCC RNA-seq data were used as the input. Quantile normalization was applied prior to deconvolution (QN = TRUE). The resulting matrix of 22 immune cell fractions was used for all subsequent clustering, differential expression, and survival analyses. Immune cell fractions were compared across anatomical subsites, including the larynx, tongue, oral floor, and other sites. Subsequent in-depth analyses were restricted to LSCC samples (n = 64).

### Dimensionality reduction and clustering of LSCC immune profiles

To characterize immune microenvironmental heterogeneity within LSCCs, the 22 CIBERSORT-derived immune cell fractions were used as input features for dimensionality reduction and unsupervised clustering. Using the R package Rtsne (v0.17), t-distributed stochastic neighbor embedding (t-SNE) was performed to visualize the high-dimensional immune landscape in two dimensions. k-means clustering was applied to the original 22-dimensional immune fraction matrix.

### Differential gene expression and pathway analysis

Analyses of differentially expressed genes (DEGs) comparing Cluster 1 (immune-desert) and Clusters 2 & 3 (immune-active) were performed using the Wilcoxon rank-sum test applied to TPM-normalized gene expression data. Prior to analysis, genes with low expression levels were removed by retaining only genes with TPM > 1 in at least 20% of samples. Fold change (FC) was calculated as the log2 ratio of mean TPM values between groups (with a pseudocount of 0.01 to avoid zero division). In addition to all-stage LSCC analyses, a supplementary DEG analysis was restricted to Stage I–III LSCC. Pathway enrichment analysis was performed using Gene Set Enrichment Analysis (GSEA). All expressed genes were ranked by log2 FC and used as input for GSEA against Gene Ontology Biological Process gene sets (pvalueCutoff = 0.05). Gene symbols were used as the key identifier type. Results were visualized as dot plots showing the top 10 enriched pathways, stratified by enrichment direction (activated vs. suppressed).

### TLS gene signature analysis

The expression levels of six established TLS-related genes-*MS4A1*, *CD79A*, *CD19*, *CXCL13*, *MZB1*, and *DERL3*-were extracted from TPM-normalized data and compared between Cluster 1 and Clusters 2 & 3 (21, 30).

### Survival analysis

OS was defined as the time from the date of initial diagnosis to the date of death from any cause or that of the last follow-up. *YWHAG* mRNA expression was dichotomized at the median into YWHAG-high and YWHAG-low groups. Survival analyses using the Kaplan–Meier method were performed in two independent cohorts (1): all LSCC samples combined (n = 64), and (2) all HNSCC samples excluding oropharyngeal carcinoma (n = 210). To assess whether YWHAG expression was an independent prognostic factor, univariate and multivariate Cox proportional hazards analyses were performed. In the LSCC cohort, covariates included age and pathological stage; in the pan-HNSCC cohort, covariates included age, sex, and pathological stage. Pathological stage was dichotomized as Stage I–II versus Stage III–IV. Hazard ratios (HR) with 95% confidence intervals (CI) were calculated. The number of covariates in multivariate models was limited to one per 10 events to avoid overfitting.

### Institutional cohort and immunohistochemistry

Formalin-fixed, paraffin-embedded tissue specimens from 23 patients with LSCC who underwent total laryngectomy at the University of Fukui Hospital between April 2013 and December 2025 were retrieved under institutional review board approval (IRB No. 20250231). All patients underwent primary total laryngectomy without prior radiation therapy or chemotherapy; salvage laryngectomy cases were excluded to avoid potential confounding effects of prior treatment on YWHAG protein expression and the tumor immune microenvironment. Immunohistochemical analysis was performed as previously reported (31). Briefly, the tissue sections (4 µm) were mounted on charged slides and dried at 60°C for 1 h. Sections were deparaffinized in xylene and rehydrated through a graded series of ethanol solutions to distilled water. Antigen retrieval was performed using heat-induced epitope retrieval in citrate buffer (pH 6.0) at 95–100°C for 10–20 min, followed by cooling at room temperature. Endogenous peroxidase activity was quenched with 3% hydrogen peroxide for 10 min. After washing, sections were incubated with a protein-blocking solution for 10–20 min at room temperature to reduce nonspecific binding.

Slides were then incubated with primary antibodies (anti-YWHAG antibody, HPA026918) at optimized dilutions (1:1000) for overnight at 4°C. After washing with PBS, sections were incubated with the EnVision+ horseradish peroxidase-labeled polymer secondary reagent for 30 min at room temperature. Immunoreactivity was visualized using 3,3′-diaminobenzidine as the chromogen, and the reaction was monitored under a microscope. Sections were counterstained with hematoxylin, dehydrated through graded ethanol and xylene solutions, and mounted with a permanent mounting medium. Appropriate positive (sections of human tonsils) and negative (sections without primary antibody) controls were included in all experiments.

Based on staining results, patients were classified as YWHAG-high (n = 14) or YWHAG-low (n = 9). Clinicopathological parameters, including age, sex, smoking burden evaluated by the Brinkman Index, tumor size, and pathological invasion (lymphatic, venous, and perineural), were compared between these two groups. In addition, OS and recurrence-free survival (RFS) were assessed in the institutional cohort. RFS was defined as the time from the date of surgery to the date of documented recurrence or that of the last follow-up in patients without recurrence. YWHAG-high and YWHAG-low groups were compared using the Kaplan–Meier method.

### Statistical analysis

The CIBERSORT algorithm was implemented using the R package CIBERSORT (v1.0, https://github.com/Moonerss/CIBERSORT) with the LM22 signature matrix. Comparisons of immune cell fractions across anatomical subsites were performed using the Kruskal–Wallis tests with Benjamini–Hochberg correction. The optimal number of clusters (k = 3) was determined using the elbow and silhouette methods, implemented using the R package factoextra. Immune cell composition across clusters was summarized and visualized using the R package pheatmap. Cluster-specific DEGs were assessed using the Wilcoxon rank-sum test with Benjamini–Hochberg correction. DEGs were defined as those with adjusted *P* < 0.1 and |log2FC| > 0.5. TLS-associated gene expression between clusters was compared using the Wilcoxon rank-sum test with Benjamini–Hochberg correction. Pathway enrichment analysis was performed using GSEA implemented in the R package clusterProfiler (v4.6.0) with the org.Hs.eg.db annotation database and a significance threshold of *P* < 0.05. OS and RFS were estimated using the Kaplan–Meier method and compared between groups using the log-rank test. In the institutional cohort, continuous variables between two groups were compared using the two-sided Mann–Whitney U test, and categorical variables were compared using Fisher’s exact test. A *P* value < 0.05 was considered significant unless otherwise specified. All statistical analyses were performed using R software (v4.1.0; R Foundation for Statistical Computing, Vienna, Austria).

## Results

### TCGA cohort characteristics and CIBERSORT deconvolution

CIBERSORT analysis was successfully applied to 210 TCGA-HNSCC samples. **Table 1** summarizes the clinical characteristics of the full HNSCC cohort. The patients in the LSCC subgroup (n = 64) were predominantly male (76.6%) and had a median age of 62.6 years, consistent with tobacco- and alcohol-related carcinogenesis (**Table 2**). Across the full HNSCC cohort, immune cell composition did not differ significantly by survival status, tumor stage, or lymph node involvement, emphasizing the need for subsite-specific analysis.

**Table 1:** Clinical and pathological characteristics of TCGA HNSCC patients.

Variable	n (%)
patients included in analysis	210 (100)
Age (mean)	60.6 years
Gender	
Male	148 (70.5)
Female	62 (29.5)
Tumor site	
Larynx	66 (31.4)
Tongue	87 (41.4)
Oral floor	27 (12.9)
Other sites	30 (14.3)
T stage	
T1	14 (6.7)
T2	59 (28.1)
T3	70 (33.3)
T4	61 (29.0)
TX	6 (28.6)
N stage	
N0	102 (48.6)
N1	42 (20.0)
N2	57 (27.1)
N3	1 (0.5)
NX	8 (3.8)
M stage	
M0	200 (95.2)
M1	3 (1.4)
MX	7 (3.3)
Stage	
I	9 (4.3)
II	43 (20.5)
III	56 (26.7)
IV	96 (45.7)

TCGA, The Cancer Genome Atlas; HNSCC, head and neck squamous cell carcinoma.

**Table 2:** Clinical characteristics of patients with LSCC according to immune clusters.

Characteristic	Cluster1 (Desert) (n = 16)	Others (n = 48)	*P* value
Age (mean, SD)	60.4 (11.5)	63.3 (8.2)	0.362
Gender (male, %)	11 (68.8)	38 (79.2)	0.498
Smoking status (n, %)			
Ever-Smoker	8 (50.0)	35 (72.9)	0.194
Never-Smoker	2 (12.5)	2 (4.2)	
No information	6 (37.5)	11 (22.9)	
cStage (n, %)			
I	1 (6.3)	1 (2.1)	0.760
II	2 (12.5)	8 (16.7)	
III	4 (25.0)	15 (31.3)	
IV	9 (56.3)	24 (50.0)	
T stage			
T1	1 (6.3)	1 (2.1)	0.627
T2	3 (18.8)	10 (20.8)	
T3	5 (31.3)	21 (43.8)	
T4	7 (43.8)	16 (33.3)	
N stage			
N0	9 (56.3)	24 (50.0)	0.616
N1	2 (12.5)	10 (20.8)	
N2	4 (25.0)	14 (29.2)	
N3	1 (6.3)	0 (0.0)	
M stage			
M0	16 (100.0)	46 (95.8)	1.000
M1	0 (0.0)	2 (4.2)	

SD, standard deviation; LSCC, laryngeal squamous cell carcinoma.

*P < 0.05, **P < 0.01, ***P < 0.001.

### LSCC exhibits unique B-cell infiltration compared with other HNSCC subsites

Kruskal-Wallis analysis with Benjamini–Hochberg correction revealed that laryngeal tumors harbored significantly higher fractions of naive B cells and plasma cells than oral floor or tongue tumors (both *P* < 0.05, **Figures 1A, B**). No other immune cell type showed enrichment specific to the larynx. This observation suggested that B cell-associated and TLS-associated transcriptional programs may merit further investigation in LSCC.

**Figure 1:**
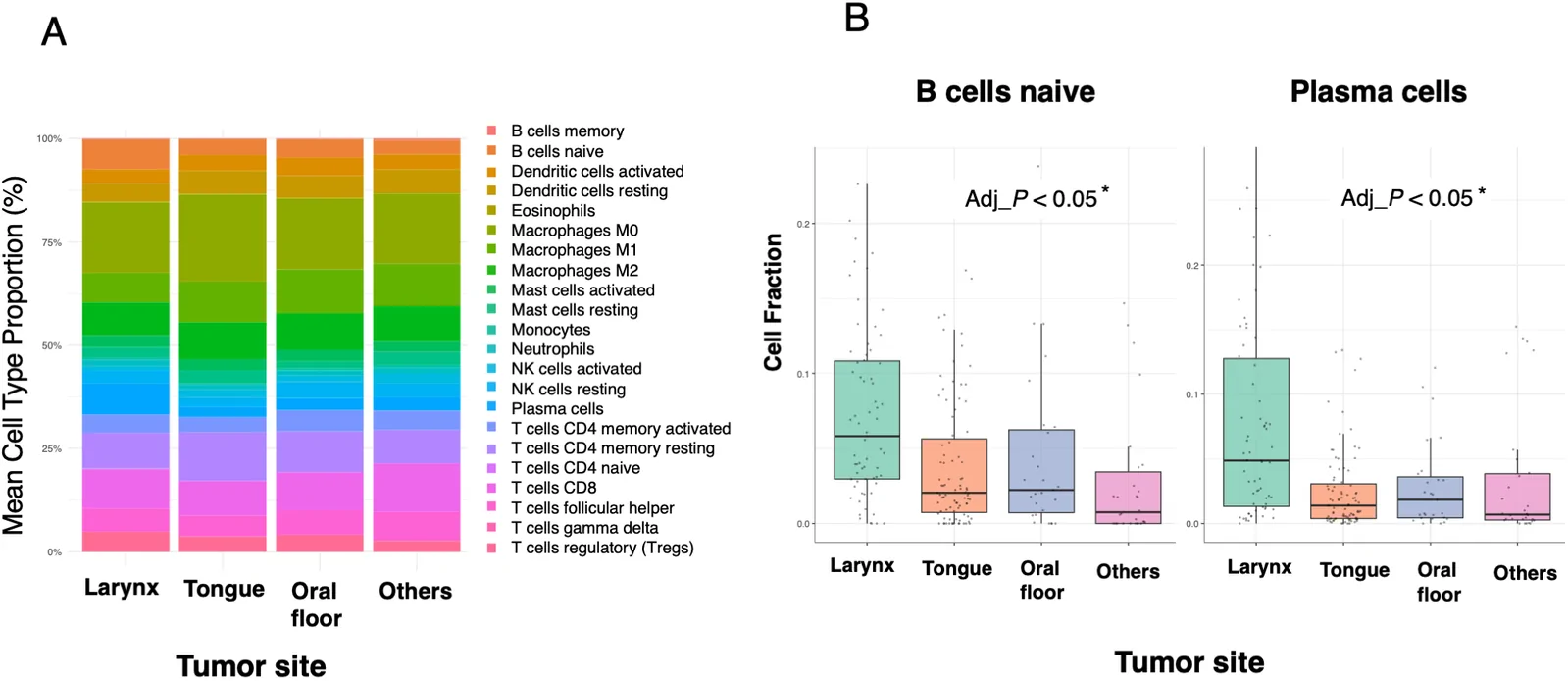
Immune cell infiltration profiles across HNSCC subsites. **(A)** Stacked bar plot illustrating the relative fractions of 22 immune cell types estimated by CIBERSORT in 210 TCGA-HNSCC samples, stratified by primary anatomical subsite (larynx, tongue, oral floor, and other sites). Each bar represents one tumor sample. **(B)** Box plots comparing the fractions of naive B cells and plasma cells across anatomical subsites. LSCC tumors exhibit significantly higher infiltration of both cell types than tumors from other subsites. Significance was determined by Kruskal–Wallis test with Benjamini–Hochberg correction (* adjusted *P* < 0.05).

### Consensus clustering defines three immune phenotypes in LSCC

Consensus clustering of immune profiles from the 64 LSCC samples identified three distinct, reproducible clusters (**Figures 2A, B**):

**Figure 2:**
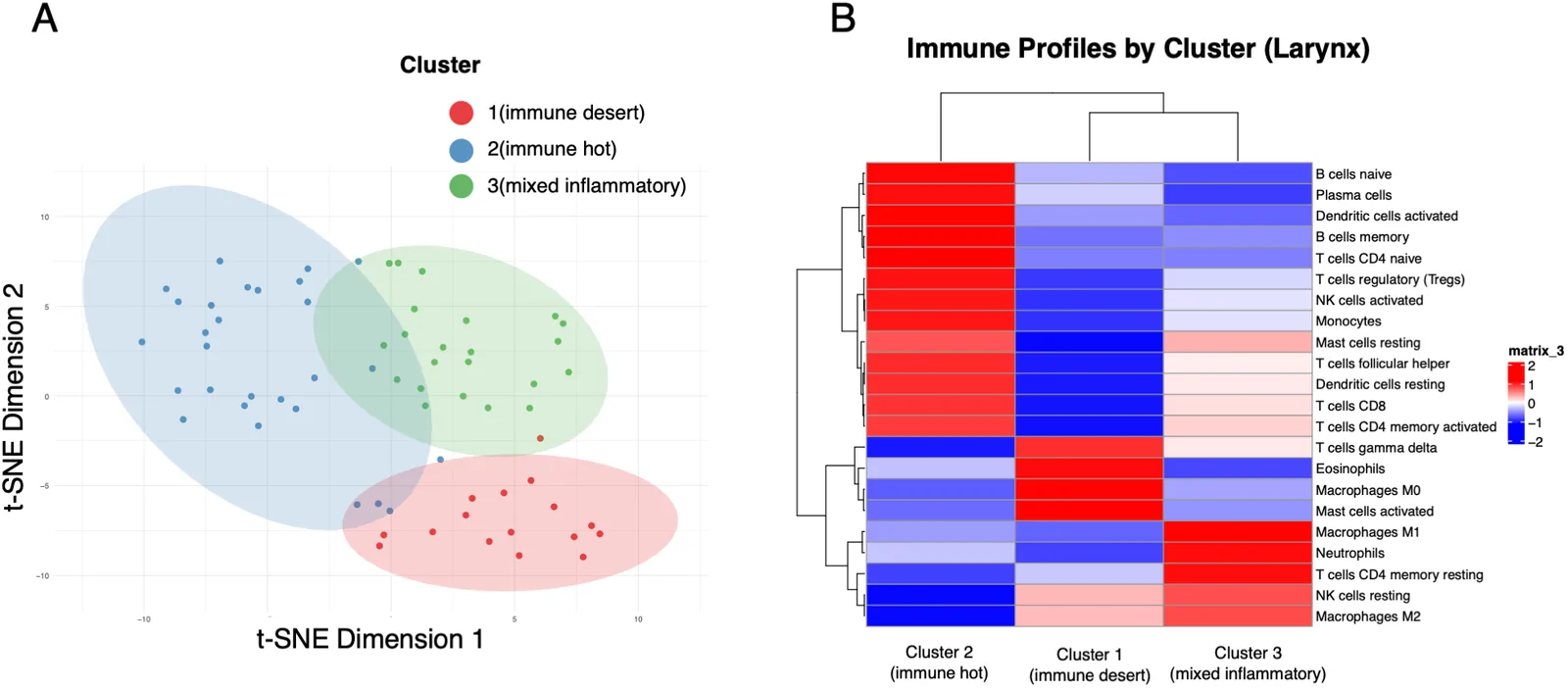
Unsupervised clustering of immune profiles in LSCC. **(A)** t-SNE visualization of 64 LSCC samples based on 22 immune cell fractions derived from CIBERSORT. Each point represents one tumor sample, colored by k-means cluster assignment (k = 3). **(B)** Heatmap depicting the mean fractions of 22 immune cell types across the three clusters (row-scaled). Cluster 1 (immune-desert) is dominated by M0 macrophages and eosinophils; Cluster 2 (immune-hot) is enriched for B cells, plasma cells, and CD8+ T cells; Cluster 3 (mixed-inflammatory) shows elevated levels of neutrophils and M1/M2 macrophages.

- Cluster 1 (immune-desert) exhibited the lowest total immune cell fraction among the three clusters (*P* < 0.001), with predominance of M0 macrophages and eosinophils, while adaptive immune effectors, including CD8+ T cells and B cells, were nearly absent. This quantitative depletion of both innate and adaptive immune populations is consistent with the immune-desert phenotype as defined by Chen and Mellman (32).
- Cluster 2 (immune-hot) markedly enriched for B cells, plasma cells, and CD8+ T cells; consistent with an immunologically active, T/B cell-inflamed phenotype.
- Cluster 3 (mixed-inflammatory) characterized by elevated counts of neutrophils and M1/M2 macrophages, reflecting coexisting innate inflammation and adaptive immune activity.

This classification recapitulates established immune contexture subtypes in solid tumors and may provide a framework for further investigation of immune heterogeneity in LSCC.

### TLS-related gene expression is abrogated in cluster 1

To validate the immune cluster classification at the transcriptomic level, we examined six established TLS-associated genes. All six-*MS4A1* (*CD20*), *CD79A*, *CD19*, *CXCL13*, *MZB1*, and *DERL3*-were significantly downregulated in Cluster 1 compared with their expression levels in Clusters 2 & 3 (adjusted *P* < 0.05, **Figure 3A**). Notably, *CXCL13* showed the most pronounced difference (adjusted *P* = 0.005), consistent with its key role in recruiting B cells and initiating TLS formation. Higher MZB1 and DERL3 expression in Clusters 2 and 3 is consistent with plasma-cell-associated transcriptional activity but does not directly demonstrate B-cell localization, functional activation, or TLS formation.

**Figure 3:**
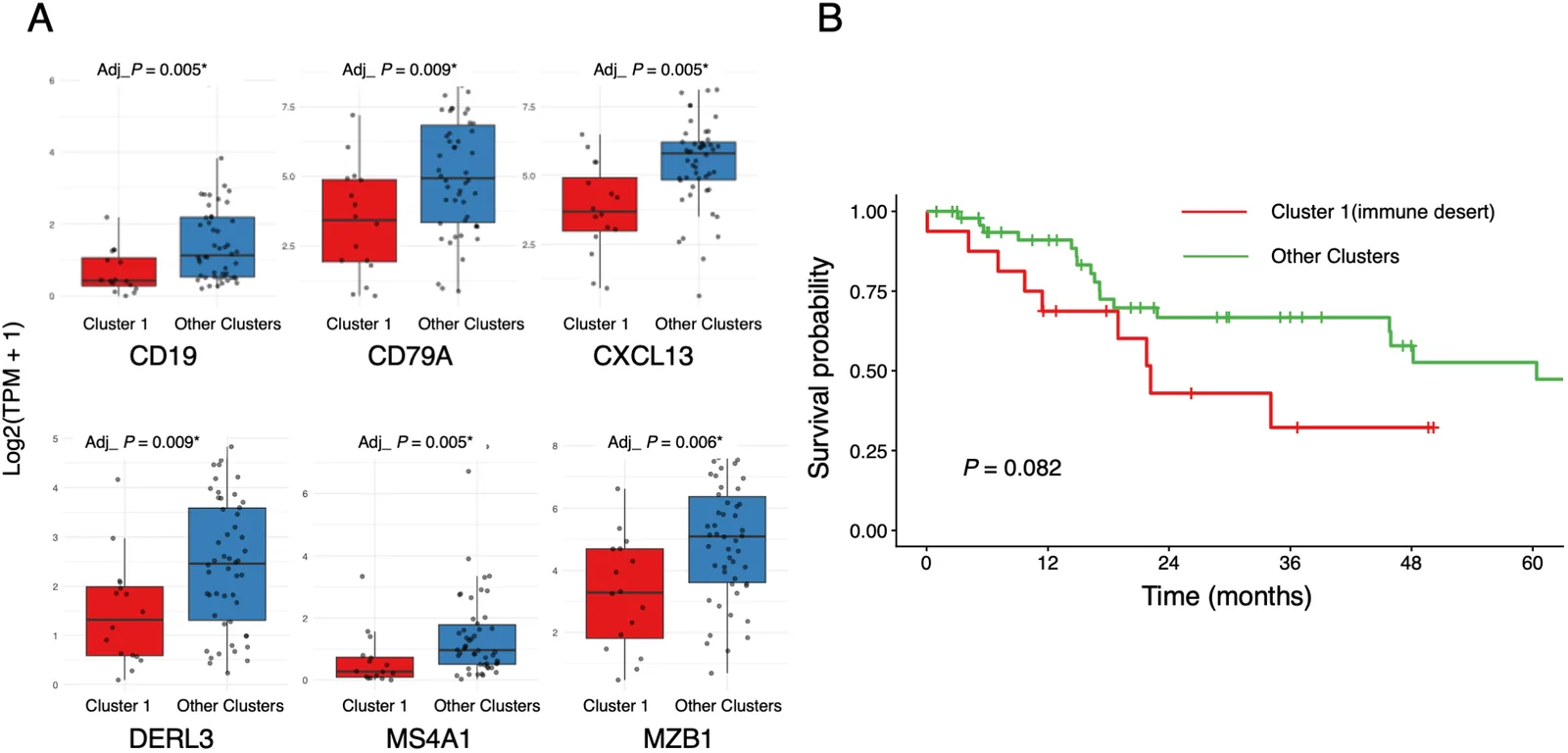
TLS-related gene expression by immune cluster and survival analysis. **(A)** Box plots comparing the expression levels (TPM) of six established TLS-associated genes-*MS4A1*, *CD79A*, *CD19*, *CXCL13*, *MZB1*, and *DERL3*-between Cluster 1 and Clusters 2 & 3. All six genes were significantly downregulated in Cluster 1 (* adjusted *P* < 0.05, Wilcoxon rank-sum test with Benjamini–Hochberg correction). **(B)** Kaplan–Meier curves for overall survival, comparing Cluster 1 (immune-desert) versus Clusters 2 & 3 (immune-active) in patients with LSCC from the TCGA cohort (log-rank *P* = 0.082).

### Cluster 1 is associated with a trend toward inferior OS

Next, we evaluated the prognostic relevance of the identified immune phenotypes in LSCC. Initially, Kaplan–Meier analysis comparing all three individual clusters (Clusters 1, 2, and 3) revealed no significant differences in OS among the groups (log-rank *P* = 0.206). Given that Cluster 1 (immune-desert) uniquely lacked active immune machinery, whereas Clusters 2 and 3 both exhibited distinct immune cell infiltration, we subsequently conducted a secondary exploratory analysis pooling Clusters 2 & 3 to compare them with Cluster 1.

In this comparison, patients in Cluster 1 had a numerically shorter median OS than those in the pooled cohort of Clusters 2 & 3, although this difference did not reach significance (log-rank *P* = 0.082, **Figure 3B**). While the lack of formal significance, potentially due to the modest sample size of this LSCC-specific TCGA subcohort, precludes definitive conclusions regarding prognostic value, the persistent numerical separation of the survival curves throughout the follow-up period warrants further validation in larger, independent patient cohorts.

### *YWHAG* is preferentially upregulated in the early-stage immune-desert cluster

DEG analysis comparing Cluster 1 versus Clusters 2 & 3 across all LSCC stages identified 11 genes significantly upregulated in Cluster 1 (immune-desert) and 161 genes significantly upregulated in Clusters 2 & 3 (**Figure 4A**). The 11 genes upregulated in Cluster 1 included *CCDC91*, *CD109*, *MMP9*, *NEFL*, *PTHLH*, *RABGEF1*, RPS3A, *SIAH1*, *SPP1*, *STK17A*, and *YWHAG*. Pathway enrichment analysis using GSEA revealed that B-cell receptor signaling, immunoglobulin production, B cell-mediated immunity, and broader lymphocyte- and leukocyte-mediated immune processes were significantly enriched in Clusters 2 & 3, confirming their immune-active phenotype. Conversely, Cluster 1 showed enrichment of non-immune pathways, including cytoplasmic translation, collagen fibril organization, connective tissue development, and cell-substrate junction organization, suggesting a microenvironmental shift toward stromal remodeling and translational upregulation in immune-desert tumors (**Figure 4B**). A supplementary DEG analysis restricted to Stage I–III LSCC-performed with a relaxed significance threshold (adjusted *P* < 0.1) to account for the small sample size-revealed *YWHAG* as the sole gene significantly upregulated in Cluster 1 (**Figures 5A, B**). This exploratory analysis identified an association between *YWHAG* overexpression and the immune-desert phenotype in early-stage LSCC.

**Figure 4:**
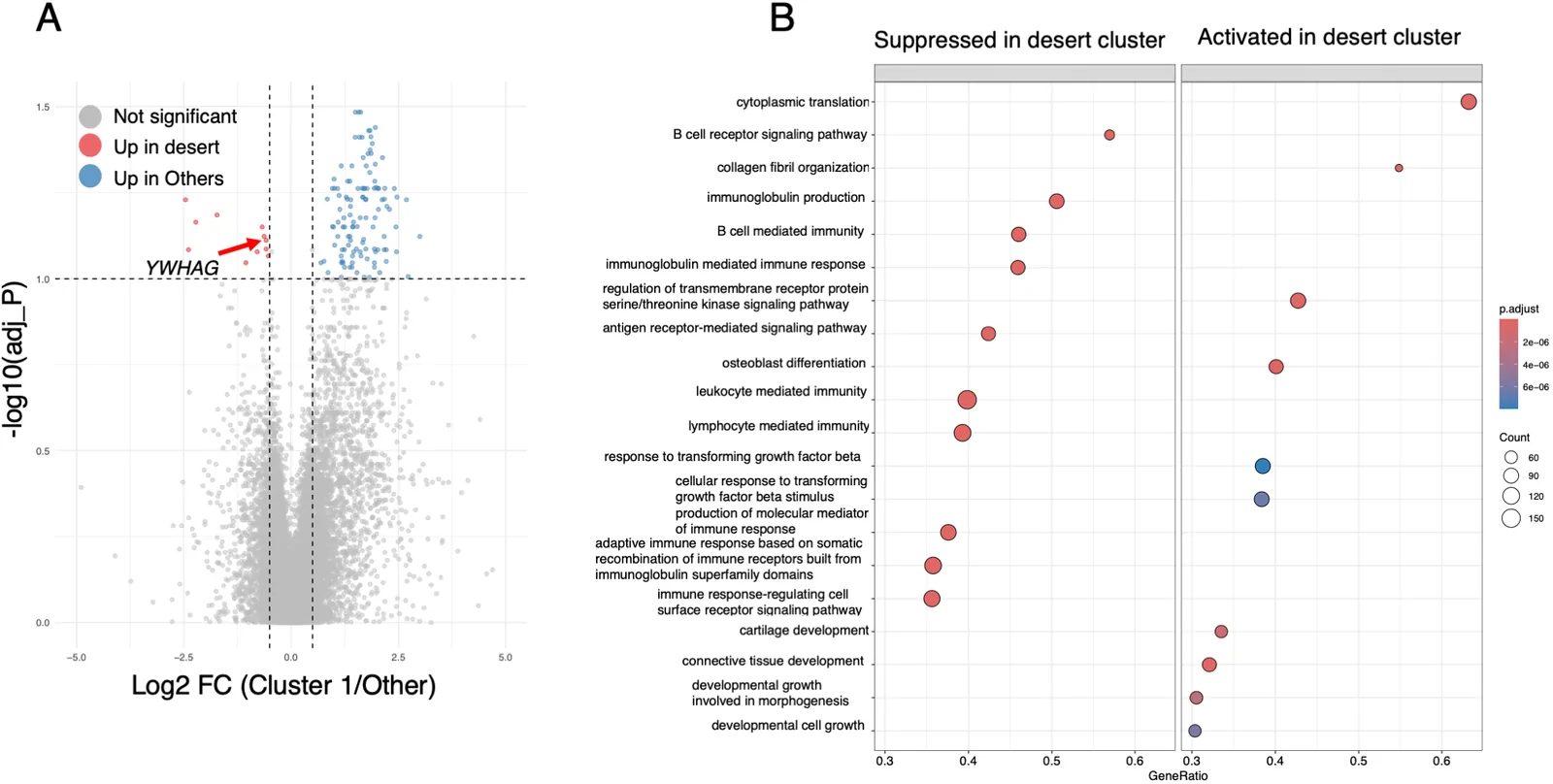
Analyses of DEGs and pathway enrichment across all LSCC stages. **(A)** Volcano plot of DEG analysis comparing Cluster 1 (immune-desert) versus Clusters 2 & 3 (immune-active) across all LSCC stages (n = 64). Genes with adjusted *P* < 0.1 and |log2FC| > 0.5 are highlighted; red indicates genes upregulated in Cluster 1 (n = 11, including *YWHAG*), and blue indicates genes upregulated in Clusters 2 & 3 (n = 161). Dashed lines indicate significance thresholds. **(B)** Dot plot of GSEA results showing the top 10 enriched Gene Ontology Biological Process (GO BP) pathways, stratified by enrichment direction. Activated pathways (enriched in Clusters 2 & 3) include B-cell receptor signaling, immunoglobulin production, and lymphocyte-mediated immunity. Suppressed pathways (enriched in Cluster 1) include cytoplasmic translation, collagen fibril organization, and connective tissue development. Dot size indicates gene count; color indicates adjusted *P*-value.

**Figure 5:**
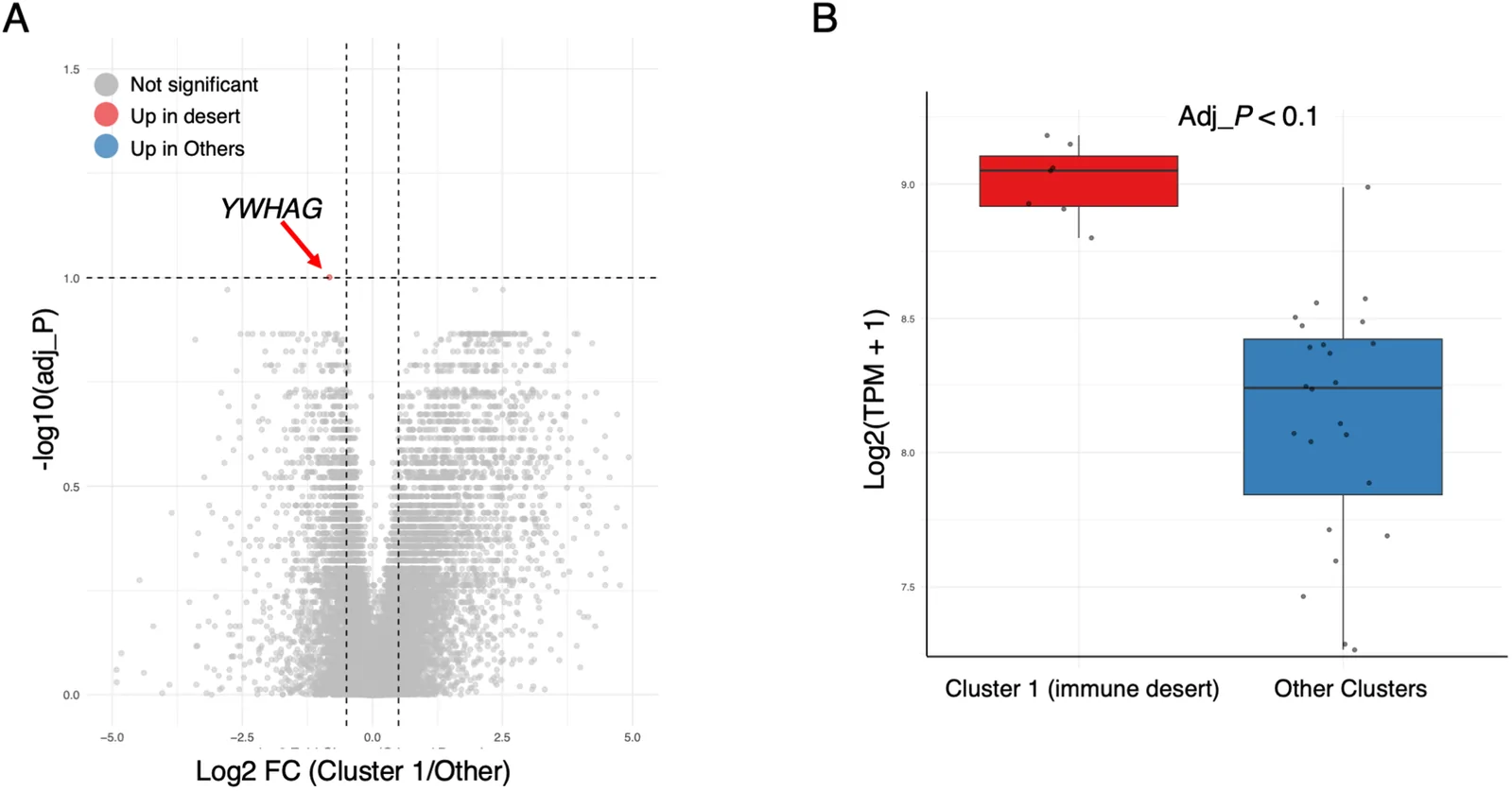
DEG analysis restricted to Stage I–III LSCC and *YWHAG* expression. **(A)** Volcano plot of DEG analysis comparing Cluster 1 versus Clusters 2 & 3 restricted to Stage I–III LSCC. *YWHAG* is identified as the sole gene significantly upregulated in Cluster 1 (adjusted *P* < 0.1, |log2FC| > 0.5). **(B)** Box plot comparing *YWHAG* mRNA expression (TPM) between Cluster 1 and Clusters 2 & 3 in Stage I–III LSCC samples. *YWHAG* expression is significantly higher in Cluster 1 (* adjusted *P* < 0.1, Wilcoxon rank-sum test).

### *YWHAG* is broadly expressed in normal tissues but enriched in malignant epithelial cells in LSCC

To further characterize the tissue and cellular distribution of *YWHAG* expression, we analyzed the Human Protein Atlas (HPA) consensus RNA expression dataset and a publicly available LSCC scRNA-seq dataset (GSE150321). HPA analysis demonstrated that *YWHAG* was broadly expressed across normal human tissues, with relatively high expression in tongue mucosa (222.8 nTPM), used as a proxy for normal squamous mucosa (**Supplementary Figure 1A**). In the LSCC scRNA-seq dataset, *YWHAG* expression was highest in malignant epithelial cells (mean normalized expression, 0.70), followed by endothelial cells (0.53), fibroblasts (0.47), and immune cells (0.37) (**Supplementary Figure 1B**). To investigate the cellular source of *YWHAG* expression in LSCC, we reanalyzed the publicly available scRNA-seq dataset GSE150321, comprising two treatment-naïve LSCC tumor specimens. Unsupervised clustering identified 18 transcriptionally distinct clusters, which were annotated as malignant/epithelial cells, T cells, myeloid cells, B cells, fibroblasts, and endothelial cells based on canonical lineage markers. *YWHAG* expression was highest in malignant epithelial cell clusters, particularly in clusters exhibiting a strong keratinocyte-like differentiation signature, and was also detected at moderate levels in fibroblast, endothelial, and myeloid populations. In contrast, *YWHAG* expression was minimal or undetectable in well-defined T- and B-cell clusters. A small number of lymphocyte clusters showed low-level *YWHAG* signals; however, these clusters had relatively low sequencing depth, and these signals were therefore interpreted with caution. Taken together, these analyses indicate that *YWHAG* is not a tumor-restricted gene and is expressed at appreciable levels in several normal tissues, including squamous mucosa; however, within LSCC tumors, its expression is enriched in malignant epithelial cells.

### High YWHAG mRNA expression independently predicts shorter OS in LSCC and HNSCC

Kaplan–Meier analysis dichotomizing patients by median *YWHAG* mRNA expression demonstrated significantly shorter OS in the YWHAG-high group in both cohorts. In the pan-HNSCC cohort excluding oropharyngeal carcinoma (n = 210), YWHAG-high patients showed significantly inferior OS than YWHAG-low patients (log-rank *P* = 0.046, **Figure 6A**). Consistent with this observation, in the LSCC-specific cohort (n = 64), YWHAG-high expression was also significantly associated with shorter OS (log-rank *P* = 0.032; **Figure 6B**). The concordant prognostic signal across both the broader HNSCC cohort and the laryngeal-specific subgroup supports further evaluation of YWHAG as a candidate prognostic marker in LSCC and HNSCC. To further evaluate whether YWHAG expression independently predicted OS after adjusting for clinical confounders, univariate and multivariate Cox proportional hazards analyses were performed in both cohorts (**Table 3**). In the LSCC cohort, univariate analysis confirmed that YWHAG-high expression was significantly associated with worse OS (HR = 2.28, 95% CI 1.05–4.97, *P* = 0.037). Multivariate analysis incorporating age and pathological stage demonstrated that YWHAG-high expression remained an independent predictor of inferior OS (HR = 2.33, 95% CI 1.06–5.08, *P* = 0.034). Consistent findings were observed in the HNSCC cohort, where YWHAG-high expression was significantly associated with worse OS in both univariate (HR = 1.58, 95% CI 1.00–2.47, *P* = 0.048) and multivariate analyses adjusting for age, sex, and pathological stage (HR = 1.58, 95% CI 1.01–2.49, *P* = 0.047). These results indicate that high YWHAG mRNA expression was independently associated with shorter OS in both TCGA cohorts.

**Figure 6:**
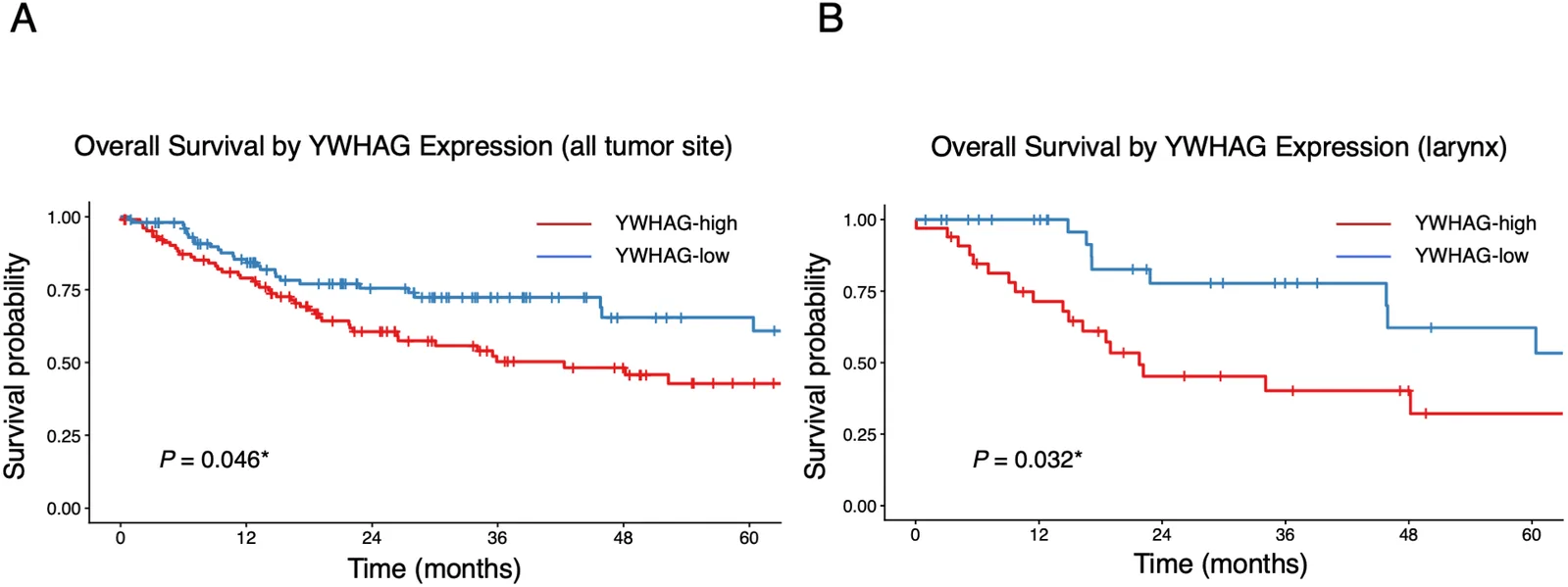
*YWHAG* mRNA expression and overall survival in TCGA cohorts. **(A)** Kaplan–Meier curves for overall survival, comparing YWHAG-high versus YWHAG-low groups in the pan-HNSCC cohort excluding oropharyngeal carcinoma (n = 210). *YWHAG* expression was dichotomized at the median value. YWHAG-high patients have significantly shorter OS (log-rank *P* = 0.046). **(B)** Kaplan–Meier curves for overall survival, comparing YWHAG-high versus YWHAG-low groups in the LSCC-specific cohort (n = 64). YWHAG-high expression is significantly associated with shorter OS (log-rank *P* = 0.032).

**Table 3:** Univariate and multivariate Cox proportional hazards analyses for overall survival.

Variable	Univariate HR (95% CI)	P value	Multivariate HR (95% CI)	P value
LSCC cohort (n = 64, events = 29)				
Age	1.02 (0.97–1.07)	0.435	1.02 (0.98–1.07)	0.409
Sex (male vs. female)	0.20 (0.09–0.49)	<0.001*	–	–
Stage (III–IV vs. I–II)	0.68 (0.27–1.68)	0.400	0.79 (0.31–2.00)	0.612
YWHAG (high vs. low)	2.28 (1.05–4.97)	0.032	2.33 (1.06–5.08)	0.034*
HNSCC cohort (n = 210, events = 80)				
Age	1.02 (1.00–1.04)	0.094	1.02 (0.995–1.04)	0.138
Sex (male vs. female)	0.63 (0.40–1.00)	0.052	0.637 (0.394–1.03)	0.066
Stage (III–IV vs. I–II)	1.11 (0.66–1.86)	0.703	1.21 (0.711–2.05)	0.485
YWHAG (high vs. low)	1.58 (1.00–2.47)	0.046*	1.58 (1.01–2.49)	0.047*

LSCC, laryngeal squamous cell carcinoma, HNSCC, head and neck squamous cell carcinoma, HR, hazard ratio, CI, confidence interval. Multivariate model includes age, sex, pathological stage, and YWHAG expression. *P < 0.05.

### YWHAG-high/CD8-low tumors are associated with poor overall survival in LSCC

To further examine the relationship between YWHAG expression and the tumor immune microenvironment, we compared the infiltration of 22 CIBERSORT-derived immune-cell subsets between YWHAG-high and YWHAG-low tumors. Among effector immune-cell populations, CD8+ T cells showed the most significant difference between the two groups (Mann–Whitney p = 0.007) and were therefore selected for subgroup survival analysis. Patients were stratified according to YWHAG expression and CD8+ T-cell infiltration using median cutoffs. Patients with a YWHAG-high/CD8-low phenotype had a higher mortality rate than the remaining patients (14/20, 70.0% vs. 15/44, 34.1%; odds ratio = 4.5) and significantly shorter overall survival (log-rank χ² = 5.47, p = 0.019). Consistently, Cox regression analysis showed that the YWHAG-high/CD8-low phenotype was associated with an increased risk of death (HR = 2.36, 95% CI 1.12–4.97, p = 0.023) (**Supplementary Figure 3**).

### YWHAG-high tumors exhibit significantly larger tumor size

To assess YWHAG protein expression in an independent institutional cohort, we enrolled an additional 23 patients who underwent total laryngectomy at our institution between April 2013 and December 2025. **Table 4** summarizes their clinical characteristics. The cohort was predominantly male (87.0%) and had a median age of 71 years (range 54–85). Pathological stages included Stage II (n = 1, 4.3%), Stage III (n = 11, 47.8%), and Stage IV (n = 11, 47.8%). During follow-up, 15 patients (65.2%) were alive, and 8 (34.8%) had died; recurrence was documented in 4 patients (17.4%). By YWHAG immunohistochemistry, 14 patients (56.5%) were classified as YWHAG-high and 9 as YWHAG-low (**Figure 7A**).

**Table 4:** Correlation between YWHAG expression and clinico-pathological parameters in LSCC.

	YWHAG_negative	YWHAG_positive	positive vs negative
Characteristic	(n = 9)	(n = 14)	*P* value
Age (mean, SD)	68.2(12.0)	71.4 (5.9)	0.409
Gender (male, %)	6 (66.7)	14 (100.0)	0.047*
BMI (mean, SD)	20.1 (3.4)	21.1 (5.1)	0.629
Smoking (BI, mean)	822.2 (419.7)	1375 (501.1)	0.012
Alcohol use (n, %)	6 (66.7)	12 (85.7)	0.343
Cancer history (n, %)	4 (44.4)	3 (21.4)	0.363
WBC (mean, SD)	5644.4 (1468.1)	6200.0 (1327.2)	0.358
Lymphocyte_rate (mean, SD)	28.2 (7.0)	24.5 (7.2)	0.326
Albumin (mean, SD)	3.7 (0.6)	3.7 (0.5)	0.789
Tumor size max(median [IQR])	20.0 [19.0, 26.0]	36.0 [30.8, 51.0]	0.003**
Tumor subsite (n, %)			
Glottic	6 (66.7)	5 (35.7)	0.329
Supraglottic	2 (22.2)	7 (50.0)	
Subglottic	1 (11.1)	2 (14.3)	
pStage (n, %)			
II	1 (11.1)	0 (0.0)	0.171
III	6 (66.7)	5 (35.7)	
IV	2 (22.2)	9 (64.2)	
pT (n, %)			
2	1 (11.1)	0 (0.0)	0.140
3	6 (66.7)	6 (42.9)	
4	2 (22.2)	8 (57.1)	
pN (n, %)			
0	8 (88.9)	8 (57.1)	0.170
1	0 (0.0)	4 (28.6)	
2	0 (0.0)	1 (7.1)	
3	0 (0.0)	1 (7.1)	
ly (n, %)			
0	7 (77.8)	8 (57.1)	0.386
1	2 (22.2)	3 (21.4)	
2	0 (0.0)	3 (21.4)	
pn			
0	7 (77.8)	7 (50)	0.340
1	1 (11.1)	5 (35.7)	
2	1 (11.1)	2 (14.3)	
V			
0	8 (88.9)	8 (57.1)	0.340
1	1 (11.1)	5 (35.7)	
2	0 (0.0)	1 (7.1)	
Adjuvant therapy (n, %)			
CRT	1 (11.1)	5 (35.7)	0.340
Recurrence (n, %)	0 (0.0)	4 (28.6)	0.127
Recurrence treatment (n, %)			
RT	0 (0.0)	1 (7.1)	
CRT	0 (0.0)	1 (7.1)	
chemotherapy	0 (0.0)	1 (7.1)	
Best supportive care	0 (0.0)	1 (7.1)	

SD, standard deviation; IQR, interquartile range, BMI, body mass index; WBC, white blood cell.

LSCC, laryngeal squamous cell carcinoma; CRT, chemo-radiation therapy; RT, radiation therapy.

*P < 0.05, **P < 0.01, ***P < 0.001.

**Figure 7:**
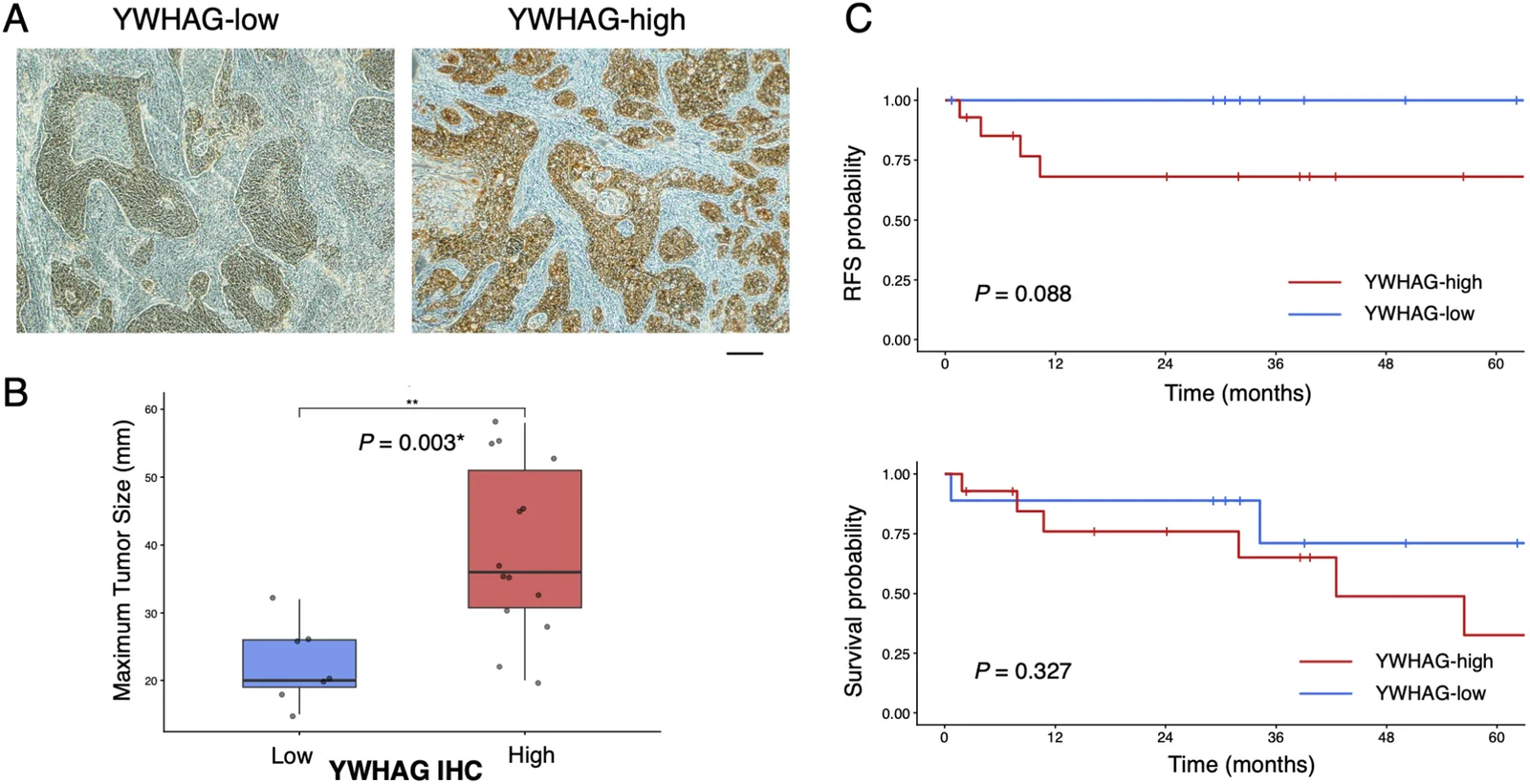
Institutional immunohistochemical (IHC) validation of YWHAG protein expression in LSCC. **(A)** Representative photomicrographs of YWHAG immunohistochemistry in YWHAG-low (left) and YWHAG-high (right) LSCC specimens. Scale bar = 200 µm. YWHAG immunoreactivity was predominantly cytoplasmic. **(B)** Box plot comparing maximum tumor size (mm) between YWHAG-low (n = 9) and YWHAG-high (n = 14) groups. YWHAG-high tumors had a significantly larger tumor size (median 36.0 mm vs. 20.0 mm; Mann–Whitney U test, *P* = 0.003). **(C)** Kaplan–Meier curves for RFS (upper) and OS (lower) comparing YWHAG-high versus YWHAG-low groups in the institutional cohort. Although neither difference reaches significance (RFS: log-rank *P* = 0.088; OS: log-rank *P* = 0.327), YWHAG-high patients show a consistent trend toward inferior survival across both endpoints.

The clinicopathological parameters were compared between YWHAG-high (n = 14) and YWHAG-low (n = 9) groups in **Table 4**. YWHAG-high tumors had a significantly larger maximum tumor size than YWHAG-low tumors (median [interquartile range], 36.0 [30.8–51.0] mm vs. 20.0 [19.0–26.0] mm, *P* = 0.003; **Figure 7B**). Additionally, smoking burden, as measured by the Brinkman Index, was significantly higher in the YWHAG-high group (mean 1375 vs. 822.2, *P* = 0.012). A significant difference in sex distribution was also observed, with all YWHAG-high patients being male (100% vs. 66.7%; *P* = 0.047). Although YWHAG-high tumors tended toward more advanced pathological stage (Stage IV: 64.2% vs. 22.2%) and higher pT and pN categories, these differences did not reach significance (*P* = 0.171, 0.140, and 0.170, respectively). Pathological invasion parameters, including lymphatic, perineural, and venous invasion, showed numerically higher rates in the YWHAG-high group but were not significantly different (*P* = 0.386, 0.228 and 0.340, respectively). Although neither difference reached statistical significance (RFS, log-rank *P* = 0.088; OS, log-rank *P* = 0.327; **Figure 7C**), no statistically significant survival association was observed in the institutional cohort. Therefore, this cohort did not provide independent prognostic validation of YWHAG expression.

## Discussion

This study integrates large-scale computational immune profiling of TCGA-HNSCC with institutional immunohistochemical assessment to characterize immune profiles in LSCC and examine associations between *YWHAG* expression and clinicopathological features. The principal findings are as follows (1): among HNSCC subsites, LSCC showed higher estimated fractions of naïve B cells and plasma cells (2); immune profiling identified three clusters with distinct inferred immune-cell compositions (3); the immune-desert phenotype was characterized by lower expression of TLS-associated genes and higher *YWHAG* expression; and (4) in the institutional cohort, high YWHAG protein expression was associated with larger tumor size.

The observation that LSCC exhibits significantly higher naive B-cell and plasma cell infiltration than other HNSCC subsites is a novel finding. Anatomical site-specific differences in the tumor immune microenvironment are increasingly recognized and may reflect local antigen exposure, distinct mucosal microbiomes, or site-specific tissue-resident immune populations (33, 34). Moreover, the substantial heterogeneity in B-cell infiltration within LSCCs, reflected in our three-cluster model, indicates heterogeneity in the LSCC immune landscape. This is consistent with recent evidence that tumor-infiltrating B cells in HNSCC comprise functionally divergent subsets-such as marginal zone B cells with immunomodulatory rather than purely regulatory properties-whose prognostic relevance may depend on disease stage (35).

The identification of three immune clusters among LSCCs, immune-desert, immune-hot, and mixed-inflammatory, suggests heterogeneity in the LSCC tumor microenvironment similar to that described in other solid tumors (32). Although the difference in overall survival between clusters did not reach statistical significance (*P* = 0.082), the numerical trend toward inferior survival in the immune-desert cluster was broadly consistent with observations in colorectal, lung, and bladder cancers, in which intratumoral immune-cell density has been associated with clinical outcomes (36). Cluster 1 exhibited lower expression of TLS-associated genes. Among these genes, *CXCL13*, a chemokine involved in B-cell recruitment and TLS organization, showed the largest inter-cluster difference (adjusted *P* = 0.005). These transcriptomic findings are consistent with an immune-desert-like profile but do not provide direct evidence of TLS presence, abundance, maturation, or spatial organization. GSEA showed reduced enrichment of adaptive immune pathways and enrichment of stromal-remodeling processes, including collagen fibril organization and connective tissue development. These patterns are compatible with an immune-excluded contexture described in established immune-contexture frameworks (37, 38), but they do not establish that extracellular-matrix remodeling creates a physical barrier or causes immune exclusion. The potential relevance of these patterns to treatment response requires prospective clinical and functional studies.

The discovery of *YWHAG* as the sole gene upregulated in the immune-desert cluster among Stage I–III LSCC, although emerging from a hypothesis-generating exploratory analysis with a relaxed significance threshold, carries important biological implications. The 14-3–3 family of proteins, which includes YWHAG, comprises multifunctional phosphoserine/phosphothreonine-binding scaffolds that regulate a broad spectrum of oncogenic processes (39, 40). Specifically, YWHAG (14-3–3 gamma) promotes cell survival by sequestering pro-apoptotic proteins, sustains cell cycle progression by inactivating CDC25 phosphatases, and amplifies oncogenic receptor tyrosine kinase signaling (22). In the context of immune biology, emerging evidence suggests that 14-3–3 proteins can modulate innate immune signaling pathways, including those involving NF-κB and TGF-β, which may contribute to immune exclusion (41). Because our findings are primarily based on transcriptomic and histological associations, a causal role of YWHAG in regulating TLS formation cannot be established. Functional studies using immunocompetent *in vivo* models will be required to determine whether YWHAG directly influences TLS development. Whether YWHAG directly suppresses immune cell recruitment or TLS formation in LSCC warrants dedicated functional investigation.

A causal relationship between YWHAG expression and TLS formation cannot be established from the present study. Although transcriptomic or proteomic profiling following YWHAG knockdown or overexpression may help identify signaling pathways regulated by YWHAG in LSCC cells, such *in vitro* approaches alone would not be sufficient to determine its direct role in TLS development. More advanced *in vitro* systems, including Transwell co-culture, multicellular co-culture, and tumor organoid–immune cell models, could partially reproduce specific cellular interactions involved in TLS-associated immune responses and may provide useful approaches for mechanistic investigation. However, TLS formation requires coordinated interactions among malignant, immune, stromal, and endothelial cells within the tumor microenvironment. Therefore, such systems would primarily model selected components of the TLS-associated process rather than fully reproduce TLS formation. Functional validation would ideally require manipulation of YWHAG expression in laryngeal cancer cells followed by transplantation into an immunocompetent animal model, allowing assessment of immune-cell recruitment, stromal organization, and TLS development *in vivo*. Establishment of such a model was beyond the scope of the present study and represents an important direction for future research.

The published literature on YWHAG expression in cancer is limited to malignancies outside the head and neck region (24, 42–45). *YWHAG* overexpression is associated with shorter disease-free survival and OS in non-small cell lung cancer, where it promotes resistance to platinum-based chemotherapy through modulation of the DNA damage response (28). In gastric cancer, *YWHAG* amplification is linked to lymph node metastasis and peritoneal dissemination (29). In colorectal cancer, *YWHAG* promotes tumor progression through the CTTN–Wnt/β–catenin signaling axis (46). Within the HNSCC field, Wang et al. previously identified *YWHAG* as a hub gene in protein–protein interaction networks constructed from TCGA-HNSCC gene expression data, suggesting its potential role in HNSCC pathogenesis (47). However, their analysis was limited to DEG and network topology, and they did not address the relationship between *YWHAG* expression and the tumor immune microenvironment. A recent cross-sectional study by Ramesh Kumar et al. reported, for the first time in the head and neck field, that *YWHAG* mRNA and protein expression are significantly upregulated in oral squamous cell carcinoma compared with adjacent non-tumor tissues and that high *YWHAG* expression correlates with advanced tumor stage, higher histological grade, and lymph node metastasis (48). Furthermore, functional pathway analysis in their study implicated YWHAG in MAPK cascade activation, VEGFA–VEGFR2 signaling, and regulation of autophagy-pathways known to drive aggressive tumor behavior (48). Our findings extend these observations specifically to LSCC, a subsite not previously investigated, and additionally demonstrate that *YWHAG* overexpression is preferentially associated with the immune-desert microenvironmental phenotype. In our institutional cohort, YWHAG-high tumors were significantly associated with larger maximum tumor size, suggesting that YWHAG overexpression may facilitate tumor growth and local expansion. This finding is biologically plausible given the established role of YWHAG (14-3–3 gamma) in promoting cell cycle progression and resistance to apoptosis. Although lymphatic invasion grade did not reach significance in our small institutional cohort, YWHAG-high tumors showed numerically higher rates of lymphatic invasion, a finding directionally consistent with the reported role of 14-3–3 proteins in promoting epithelial-to-mesenchymal transition and cell motility (49). Prospective validation in larger cohorts is needed to determine whether this trend reflects a true biological association.

YWHAG is not a tumor-restricted molecule, as analysis of the Human Protein Atlas demonstrated appreciable expression across several normal tissues, including squamous mucosa. Nevertheless, our scRNA-seq reanalysis showed that, within the LSCC tumor microenvironment, YWHAG expression was highest in malignant epithelial cells compared with endothelial, fibroblast, and immune-cell populations. These findings suggest that YWHAG may more appropriately be considered a tumor-associated prognostic and immune-related biomarker rather than a tumor-specific therapeutic target. Given its substantial expression in normal tissues, the feasibility of directly targeting YWHAG would require careful evaluation of potential on-target/off-tumor effects and tissue-specific toxicity. Some limitations of this study should be acknowledged. The TCGA LSCC subcohort is modest in size (n = 64), limiting statistical power; the Kaplan–Meier separation between clusters (*P* = 0.082) and the Stage I–III DEG analysis (adjusted *P* < 0.1 threshold) reflect this constraint. Furthermore, the TCGA dataset lacked detailed clinical annotations, including tumor subsite classification (glottic, supraglottic, and subglottic), primary treatment modality, and adjuvant therapy information, precluding adjustment for these clinically important variables in the survival and immune subtype analyses. Our institutional immunohistochemistry cohort is also small (n = 23 patients), and the associations with individual pathological parameters (lymphatic, perineural, and venous invasion) did not reach significance. Moreover, owing to the small sample size and limited number of events in the institutional cohort, multivariate Cox regression analysis for OS and RFS was not feasible; the prognostic associations observed in this cohort should therefore be interpreted as exploratory rather than definitive.

Another limitation of the present study is the lack of spatially resolved analysis of YWHAG expression within the tumor microenvironment. Although our scRNA-seq reanalysis indicated that YWHAG expression was highest in malignant epithelial cells, the spatial relationship between YWHAG-expressing cells, infiltrating immune-cell populations, and TLS structures could not be determined. Multiplex immunohistochemistry, multiplex immunofluorescence, or spatial transcriptomic approaches will be required to define the cellular localization of YWHAG and clarify its spatial association with immune-cell infiltration and TLS formation in LSCC. An additional limitation is that the present study did not comprehensively characterize TLS heterogeneity, including TLS maturation status, cellular composition, or the spatial organization of tumor-infiltrating B cells. Because the biological and prognostic significance of TLSs may vary according to their developmental stage and cellular architecture, TLS abundance alone may not fully capture their functional relevance within the tumor microenvironment. Indeed, tumor-infiltrating B cells can exert either pro- or anti-tumor effects depending on their phenotype and spatial context, with mature, germinal-center-containing TLSs generally associated with antitumor immunity, whereas regulatory B (Breg) cell-dominated infiltrates may instead promote immune suppression (50). Future studies incorporating detailed phenotypic and spatial characterization of TLSs and B-cell-rich immune niches will be required to clarify how these features interact with YWHAG expression and influence clinical outcomes in LSCC. Finally, the biological relationship between *YWHAG* overexpression and immune exclusion is correlative and requires experimental validation.

## Conclusion

Among HNSCC subsites, LSCC showed higher estimated fractions of naïve B cells and plasma cells. Immune profiling identified three clusters with distinct inferred immune-cell compositions, including an immune-desert phenotype characterized by lower expression of TLS-associated genes. Higher *YWHAG* mRNA expression was associated with the immune-desert phenotype, and high YWHAG protein expression was associated with larger tumor size in the institutional cohort. These exploratory associations do not establish a causal role for YWHAG in immune exclusion or TLS formation, nor do they establish YWHAG as a prognostic biomarker or therapeutic target. Larger clinical cohorts and functional studies are required to validate these findings.

## Data Availability

The original contributions presented in the study are included in the article/**Supplementary Material**. Further inquiries can be directed to the corresponding authors.
