# Supplementary material for: YWHAG overexpression correlates with an immune-desert microenvironment and poor prognosis in laryngeal squamous cell carcinoma
Source: Front Immunol. 2026 Sep 15;17:1936961. doi: 10.3389/fimmu.2026.1936961 (PMC13619832; doi:10.3389/fimmu.2026.1936961)
Supplement: Supplementary file 1 [file DataSheet1.pdf]

Supplementary Figures

Supplement Figure1

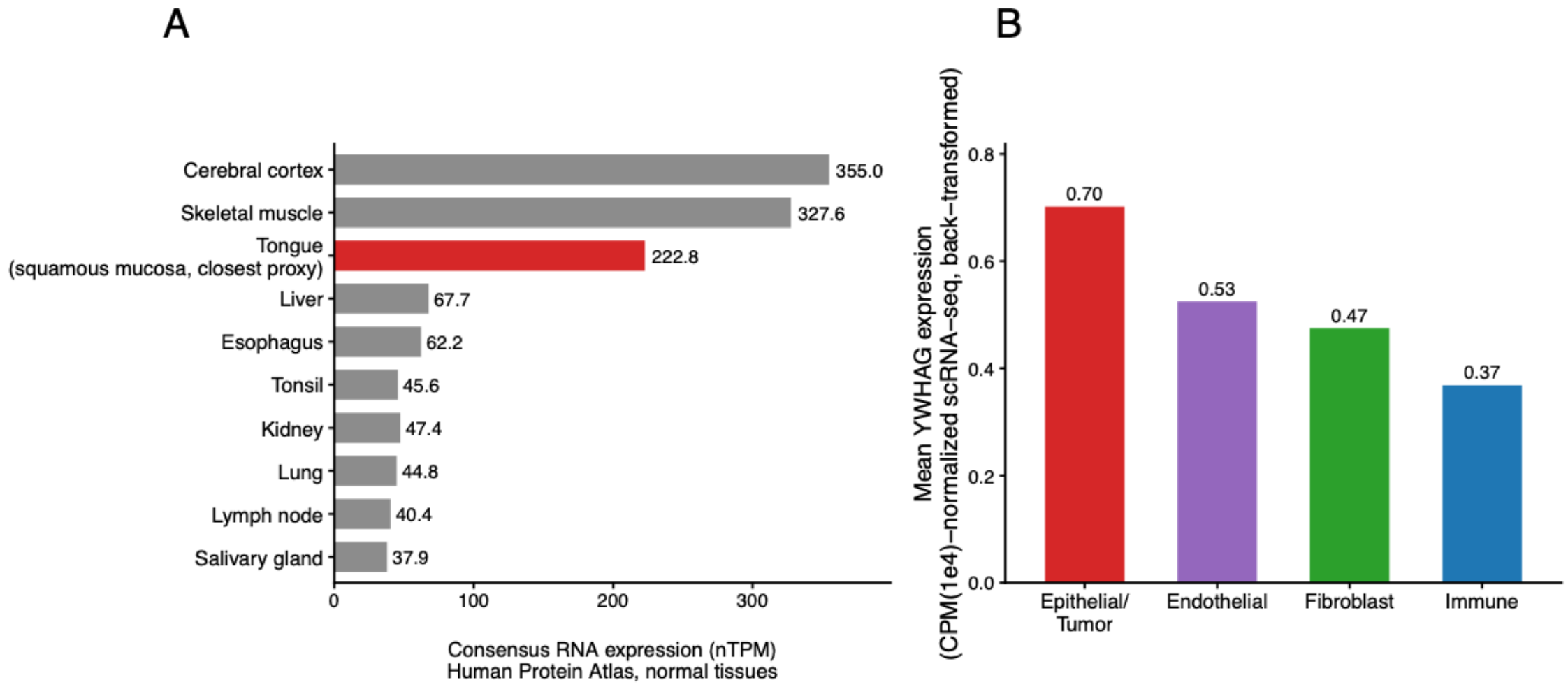

Supplementary Figure 1. YWHAG expression across normal human tissues and tumor-infiltrating cell lineages in LSCC.

(A) Bar plot of YWHAG mRNA expression (Human Protein Atlas consensus RNA dataset; nTPM, normalized transcripts per million) across representative normal human tissues. Tongue mucosa was used as the closest available proxy for laryngeal squamous epithelium, as larynx is not annotated as a discrete tissue category in the Human Protein Atlas. YWHAG showed broad expression across tissues, with the highest levels in cerebral cortex (355.0 nTPM) and skeletal muscle (327.6 nTPM), and comparatively high expression in squamous mucosa (tongue, 222.8 nTPM), exceeding that observed in esophagus (62.2 nTPM), kidney (47.4 nTPM), tonsil (45.6 nTPM), lung (44.8 nTPM), lymph node (40.4 nTPM), and salivary gland (37.9 nTPM).

(B) Mean YWHAG expression (normalized single-cell expression) across the major cell lineages identified within LSCC tumor tissue by an independent single-cell RNA-sequencing (scRNA-seq) reanalysis of the publicly available dataset GSE150321 (marker-based lineage classification; see Methods). Expression was highest in the epithelial/malignant tumor-cell lineage (mean normalized expression, 0.70), followed by endothelial cells (0.53), fibroblasts (0.47), and immune cells (0.37), indicating that within the tumor microenvironment, YWHAG expression is concentrated in the epithelial tumor-cell compartment rather than distributed uniformly across stromal and immune components.

Data in (A) are from the Human Protein Atlas (https://www.proteinatlas.org/ENSG00000170027-YWHAG/tissue, consensus RNA dataset, accessed 2026-08-08). Data in (B) are derived from a marker-based scRNA-seq lineage classification of GSE150321 (n = 3,756 epithelial/tumor, 5,913 immune, 675 fibroblast, and 586 endothelial cells; a separate, coarser classification from the Seurat-based clustering shown in Supplementary Figure 2; see Methods).

Supplement Figure2

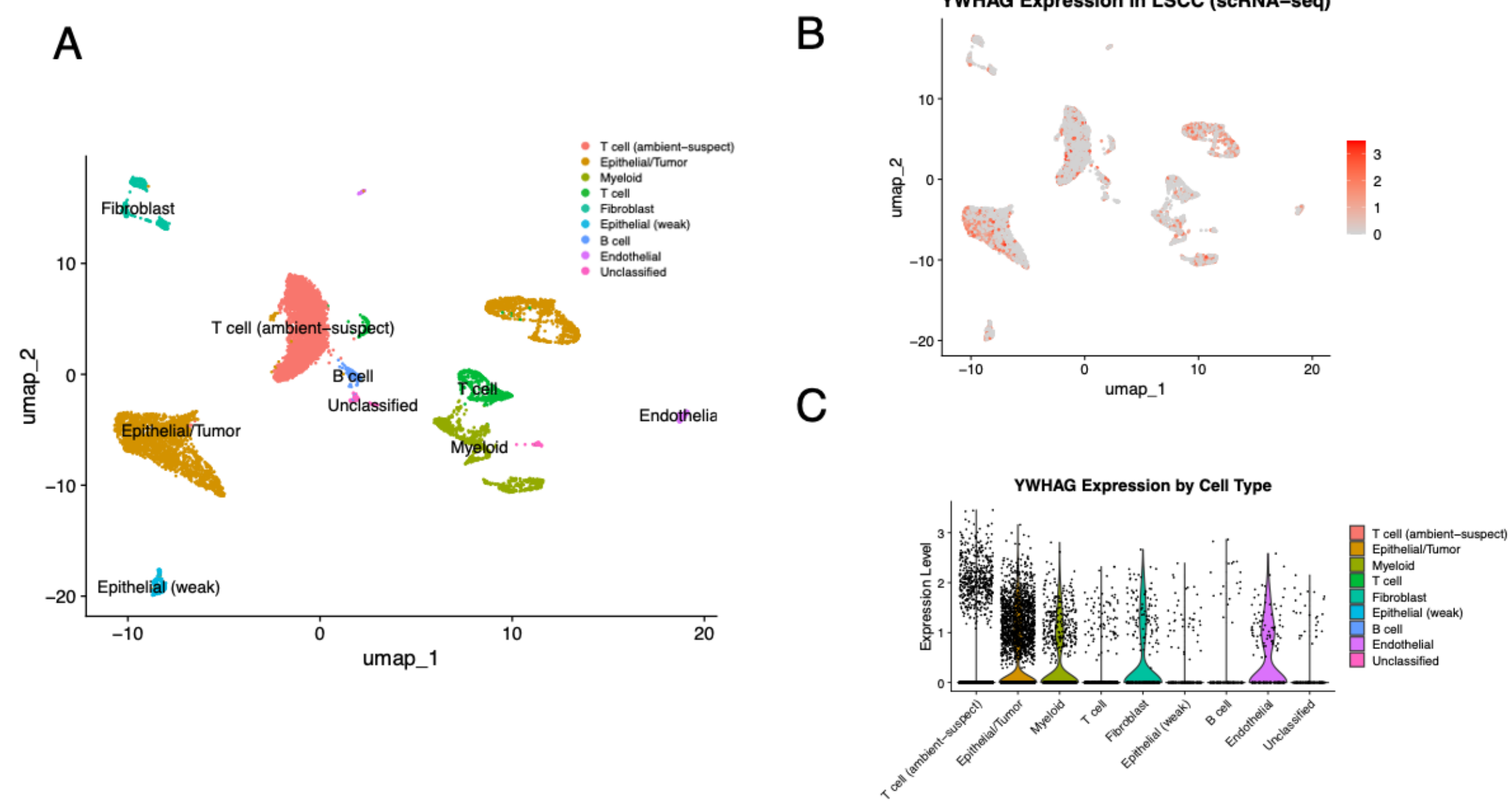

Supplementary Figure 2. Single-cell RNA-sequencing reanalysis of a publicly available LSCC dataset (GSE150321) identifies the cellular source of YWHAG expression.

(A) Uniform Manifold Approximation and Projection (UMAP) plot of 8,201 single cells from two treatment-naïve LSCC tumor specimens (GSE150321; Song et al., Int J Cancer 2020), colored by unsupervised clustering (18 transcriptionally distinct clusters, resolution = 0.4) and annotated into nine cell populations based on canonical marker gene expression: epithelial/tumor cells (n = 2,865), a minor epithelial subcluster with weak marker expression (n = 183), T cells (n = 536), a $CD3^+/PTPRC^+$ subcluster suspected of ambient RNA contamination from the surrounding epithelial/tumor population (n = 3,077; see panel C), myeloid cells (n = 756), B cells (n = 134), fibroblasts (n = 318), endothelial cells (n = 177), and unclassified cells (n = 155).

(B) UMAP feature plot (left) and violin plot (right) of YWHAG expression across annotated clusters/lineages. YWHAG was predominantly expressed in epithelial/tumor-cell clusters, with moderate expression in fibroblast, endothelial, and myeloid clusters, and minimal expression in the well-defined (non-ambient) T- and B-lymphocyte clusters.

(C) Quality-control comparison of sequencing depth (nCount_RNA and nFeature_RNA) between the $CD3^+/PTPRC^+$ subcluster showing residual YWHAG signal and the remaining well-defined lymphocyte clusters. The former showed lower sequencing depth, supporting ambient RNA contamination from the surrounding epithelial/tumor population as the likely explanation, rather than genuine lymphocyte expression of YWHAG.

Cells were filtered by nFeature_RNA (200–6,000) and mitochondrial content (<20%) prior to clustering (see Methods). Cell-lineage assignment reflects marker-gene expression; malignancy of the epithelial/tumor-cell clusters was not independently confirmed by copy-number-based methods (e.g., inferCNV), and this is noted as a limitation in the Discussion.

Supplementary Figure3

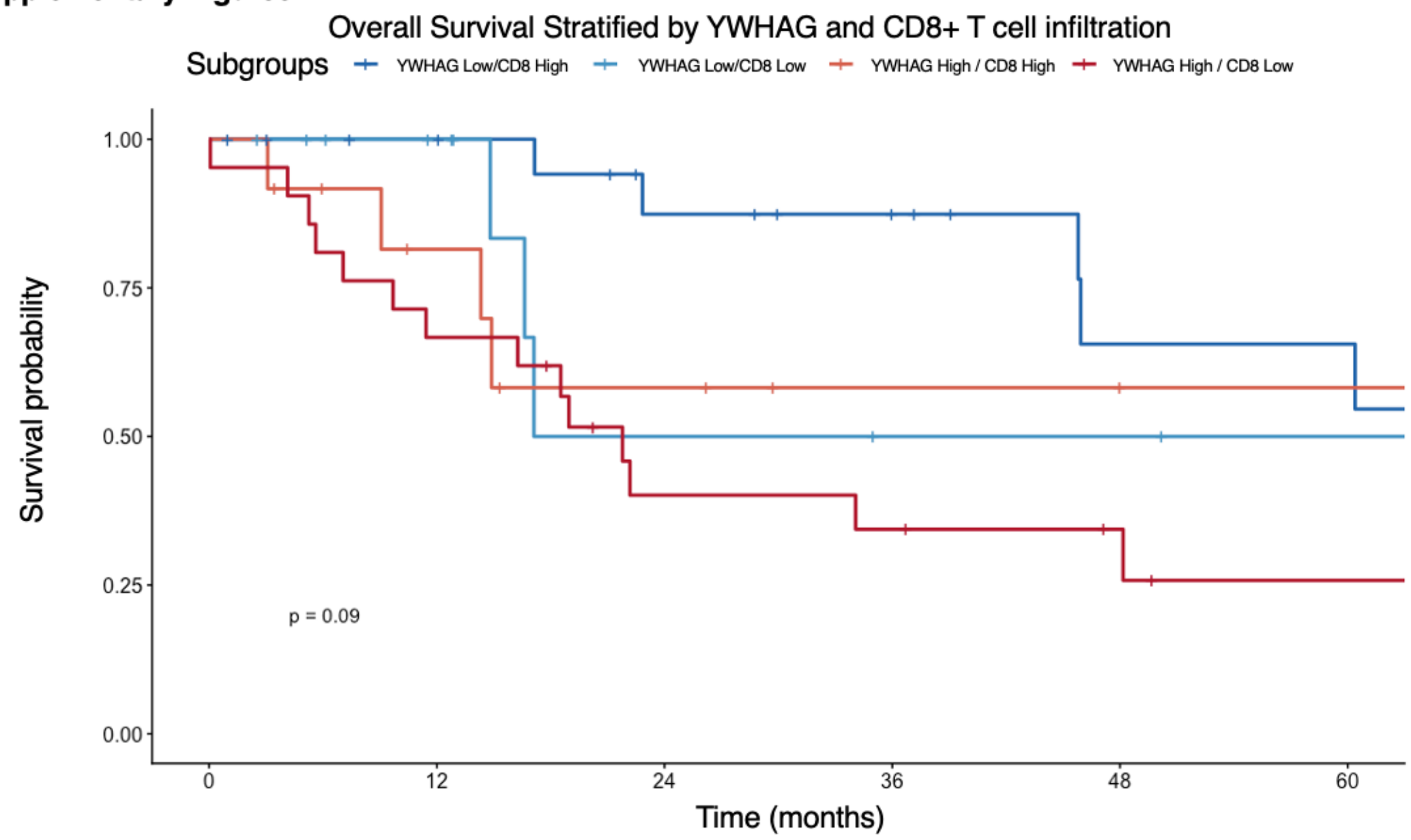

Supplementary Figure 3. Overall survival stratified by YWHAG expression and CD8⁺ T-cell infiltration in the TCGA-Larynx cohort.

Kaplan–Meier overall survival curves for four subgroups defined by YWHAG expression (high/low) and CD8⁺ T-cell infiltration (high/low), each dichotomized by a median split within the TCGA-Larynx cohort ($n = 64$; two patients lacking clinical stage annotation were excluded, consistent with the cohort used for the main survival analyses). CD8⁺ T cells were selected as the stratifying immune subset because, among the 22 CIBERSORT-estimated immune cell subsets, they showed the most robust difference between YWHAG-high and YWHAG-low tumors (Mann–Whitney U test, $p = 0.007$). The omnibus log-rank test across all four subgroups did not reach statistical significance ($\chi^2 = 7.03$, df = 3, $p = 0.09$). However, in the pre-specified, single degree-of-freedom contrast comparing the composite YWHAG-high/CD8-low phenotype ($n = 20$) against all remaining patients ($n = 44$) — the comparison most directly relevant to identifying a clinically actionable high-risk subgroup — mortality was 70.0% (14/20) versus 34.1% (15/44) (odds ratio, 4.5), overall survival was significantly shorter (log-rank test, $\chi^2 = 5.47$, $p = 0.019$), and univariate Cox proportional-hazards regression confirmed the association (HR = 2.36, 95% CI 1.12–4.97, $p = 0.023$).

Given the modest cohort size, this subgroup analysis should be interpreted as hypothesis-generating (see Discussion).
